# Stress-inducible WASF3 regulates brain metabolism and cognitive function

**DOI:** 10.1073/pnas.2617198123

**Published:** 2026-10-27

**Authors:** Abu M. Syed, Jin Ma, Ping-yuan Wang, Youlim Son, Rozhin Penjweini, Nikhil Mattu, Jay R. Knutson, Sachiko Nakamori-Yamashita, Danielle A. Springer, Ananya Ganguly, Chengyu Liu, Paul M. Hwang

**Affiliations:** ^a^ Cardiovascular Branch, National Heart, Lung, and Blood Institute, National Institutes of Health, Bethesda, MD 20892; ^b^ Laboratory of Advanced Microscopy and Biophotonics, National Heart, Lung, and Blood Institute, National Institutes of Health, Bethesda, MD 20892; ^c^ Murine Phenotyping Core, National Heart, Lung, and Blood Institute, National Institutes of Health, Bethesda, MD 20892; ^d^ Transgenic Core, National Heart, Lung, and Blood Institute, National Institutes of Health, Bethesda, MD 20892

**Keywords:** ME/CFS, mitochondria, ER stress, MAMs, ATPIF1

## Abstract

Wiskott–Aldrich syndrome protein family member 3 (WASF3) disrupts mitochondrial respiration and is induced in association with endoplasmic reticulum stress in myalgic encephalomyelitis/chronic fatigue syndrome (ME/CFS). Because cognitive impairment is a hallmark of ME/CFS, we investigated the role of WASF3 in brain metabolism and function using WASF3 transgenic and knockout mouse models. WASF3 disrupted respiratory complex IV and localized to mitochondria-associated membranes, known to modulate cellular processes important for neuronal activity. Overexpression of WASF3 in the hippocampus, a critical region for memory formation, induced a metabolic shift toward glycolysis and promoted a “hypometabolic” state. Increased WASF3 also caused a reduction in ATPIF1, previously shown to facilitate cognitive function, and impaired learning. Conversely, WASF3 deficiency increased ATPIF1 level, enhanced oxidative metabolism, and preserved cognitive function under stress. Together, these findings demonstrate that WASF3 suppresses oxidative metabolism in the brain and contributes to cognitive dysfunction, providing mechanistic insights into the “brain fog” reported in ME/CFS.

## Significance

Cognitive impairment, often described as “brain fog,” is a disabling feature of chronic fatigue syndrome (ME/CFS), but its underlying mechanism remains unclear. This study shows that stress-inducible Wiskott–Aldrich syndrome protein family member 3 (WASF3), previously linked to mitochondrial dysfunction in skeletal muscle of patients with ME/CFS, also suppresses oxidative metabolism in the brain. Increased WASF3 shifted hippocampal metabolism toward a hypometabolic state and impaired learning. Conversely, reduced WASF3 enhanced oxidative metabolic markers and protected cognitive function during cellular stress. These findings identify WASF3 as a mediator linking endoplasmic reticulum (ER) stress to brain bioenergetic dysfunction and suggest that targeting ER stress-WASF3 signaling may alleviate cognitive symptoms in ME/CFS and related postviral fatigue disorders more broadly.

Myalgic encephalomyelitis/chronic fatigue syndrome (ME/CFS) is a debilitating chronic disease characterized by multisystem symptoms such as unrelenting fatigue, postexertional malaise (PEM), immunologic abnormalities, unrefreshing sleep, and various cognitive and autonomic impairments for which there is no specific treatment (1). It is estimated to affect ~1% of the population worldwide. The cognitive symptoms, commonly referred to as “brain fog”, are complex and can be more disabling than the physical symptoms (2). The dramatic worsening of symptoms such as poor concentration and memory after physical or mental stress is referred to as PEM, a pathognomonic feature of ME/CFS that highlights involvement of the brain in this disorder (1). Although its pathogenesis is not well understood, studies have consistently reported evidence of mitochondrial dysfunction in ME/CFS (3–5). We previously identified Wiskott–Aldrich syndrome protein family member 3 (WASF3), induced by endoplasmic reticulum (ER) stress, as a mediator of mitochondrial dysfunction in ME/CFS (6), but its role in brain metabolism and function is unknown. Using genetic mouse models, we here report that WASF3 can impact mitochondrial metabolism in the brain and affect cognitive function resembling features of ME/CFS.

## Results

### Elevated WASF3 Disrupts Mitochondrial Function and Impairs Cognition.

**Fig. 1.**
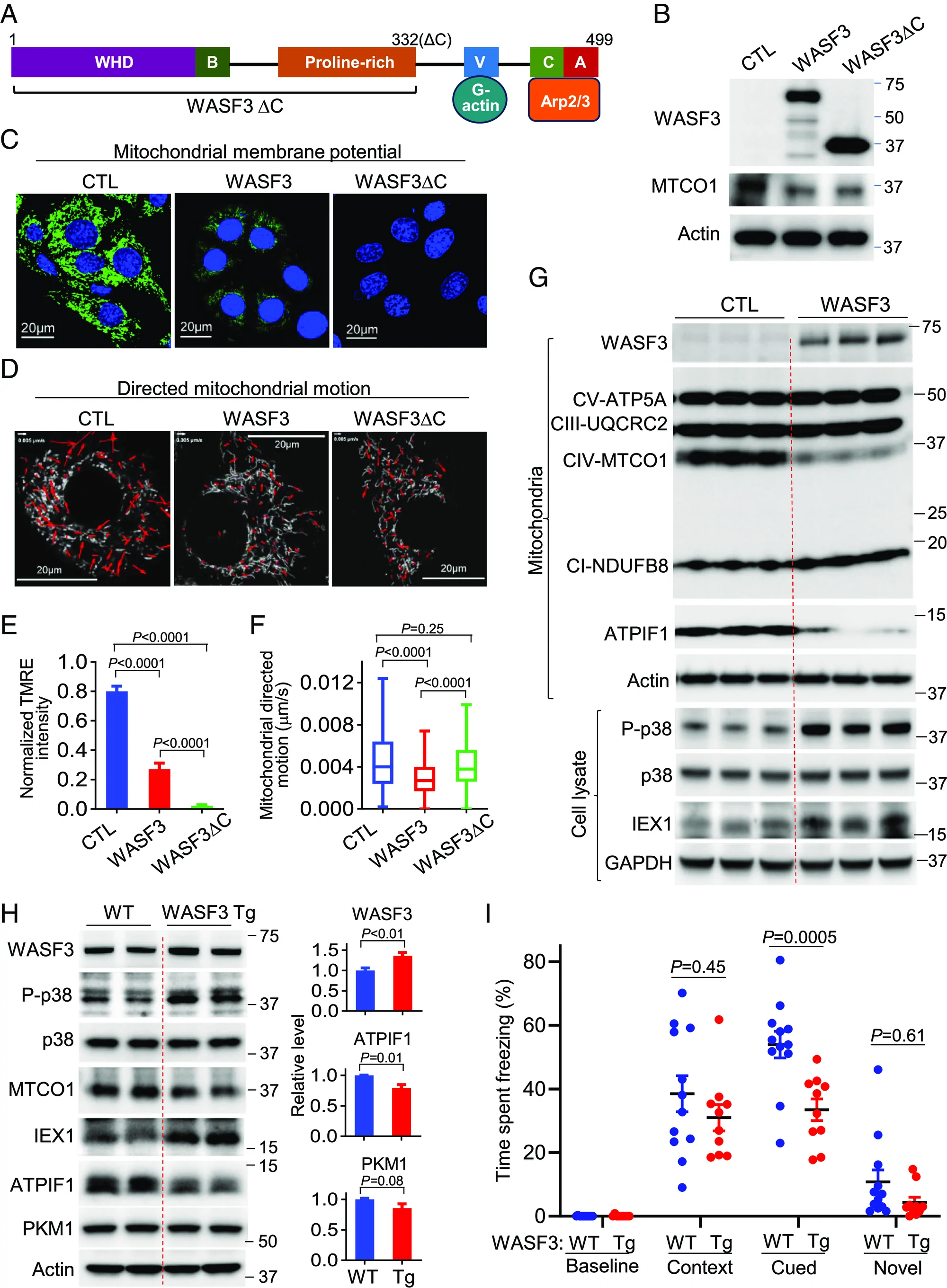
Overexpression of WASF3 disrupts mitochondrial homeostasis and impairs cognitive function. (*A*) Gene structure of WASF3; full-length form and C-terminal deletion (WASF3ΔC) construct are depicted. WASF homology (WHD), basic (B), proline-rich, verprolin (V), cofilin (C), and acidic (A) domains. Monomeric actin (G-actin) and actin regulatory protein 2 and 3 (Arp2/3). (*B*) Control (CTL) C2C12 cells, along with cells stably transfected with either full-length WASF3 (1 to 499) or the C-terminal–truncated WASF3 variant (WASF3ΔC, 1 to 332) cDNA, were immunoblotted and examined for their effect on respiratory complex IV subunit MTCO1. (*C*) Mitochondrial membrane potential was measured by staining these stably transfected cells with tetramethylrhodamine ethyl ester (TMRE). (Scale bars, 20 µm.) (*D*) Directed mitochondrial motion was measured by spatiotemporal image correlation spectroscopy (STICS). The orientation and length of the red arrows indicate the direction and magnitude of the directed motion, respectively. (*E*) Quantification of mitochondrial membrane potential (n = 10). (*F*) Quantification of directed mitochondrial motion (n = 463 to 603). (*G*) Purified mitochondria from C2C12 cells transduced with full-length WASF3 lentivirus were analyzed by immunoblotting to assess its effect on mitochondrial respiratory subunits and other indicated proteins. (*H*) Cerebral cortex tissues of wild-type (WT) and WASF3 Tg mice (1 y old males) were immunoblotted, and the indicated proteins were quantified (n = 4 to 6). (*I*) Fear conditioning was determined by measuring time spent freezing in response to cued (tone), contextual, and novel stimuli. Each group of WT and WASF3 Tg mice (~4 mo old) comprised of 5 females and 5 males (n = 10). Values are mean ± SE. Statistical difference by one-way ANOVA (*E*), Mann–Whitney U test (*F*), two-tailed Student’s *t* test (*H*), and two-way ANOVA (*I*).

WASF3 has been well studied for its role in regulating the actin cytoskeleton, but less is known about its function in mitochondria (7). WASF3 contains an N-terminal WASF homology domain (WHD) and a C-terminal verprolin-cofilin-acidic (VCA) region, which can interact with actin and the actin-related protein 2/3 complex (Arp 2/3) to regulate actin polymerization (Fig. 1*A*) (8). To further dissect its effects on the mitochondria, we overexpressed full-length WASF3 in C2C12 cells and observed loss of mitochondrial membrane potential (ΔΨ_m_), consistent with its disruption of respiration (6), as well as decreased actin-dependent directed mitochondrial motion (Fig. 1 *B*–*F*). As predicted based on its structure and known effects on the cytoskeleton (9), deletion of the C-terminal actin-interacting domain of WASF3 (WASF3ΔC) abolished its inhibition of directed mitochondrial motion (Fig. 1 *A*, *B*, *D*, and *F*), but did not affect its inhibition of respiratory complex IV (CIV) (MTCO1) and ΔΨ_m_ (Fig. 1 *B*, *C*, and *E*). These observations were consistent with a previous report that WASF3 interacts with mitochondria via insertion of its N terminus into the mitochondrial membrane (10).

Highlighting the need to maintain ΔΨ_m_ for cell survival when respiration is disrupted, WASF3 overexpressing C2C12 cells with selectively decreased CIV (MTCO1) showed markedly lower levels of ATPase inhibitory factor 1 (ATPIF1, encoded by *ATP5IF1*), a regulatory subunit of the mitochondrial ATP synthase that blocks the reverse hydrolysis of ATP for preserving ΔΨ_m_ (Fig. 1*G*) (11). Likewise, in vivo, WASF3-overexpressing transgenic (Tg) mice exhibited reduced ATPIF1 and MTCO1 levels in brain tissues compared with WT controls (Fig. 1*H*). Both in vitro and in vivo, WASF3 overexpression increased protein levels of immediate-early response 3 (IER3, also called IEX1), a protein known to target and promote ATPIF1 degradation (Fig. 1 *G* and *H*) (12). As previously reported (13), we observed that WASF3 overexpression induces activating phosphorylation of p38 mitogen-activated protein kinase (p38 MAPK), a signaling kinase known to induce IEX1 and proposed to play a central role in mediating fatigue in ME/CFS (Fig. 1 *G* and *H*) (6, 14–16). We also examined PKM1, which is highly expressed in neurons and supports oxidative metabolism (17). Quantitative analysis showed that PKM1 levels were slightly reduced in WASF3 Tg samples compared with WT controls, although the difference did not reach statistical significance (Fig. 1*H*).

The reduction in ATPIF1 levels by WASF3 overexpression correlated with the cognitive impairment of ME/CFS as ATPIF1 deficiency negatively affects memory, while ATPIF1 overexpression enhances synaptic transmission and learning (18). Accordingly, ATPIF1 has been found to be lower in patients with ME/CFS compared to healthy controls by a serum proteomics screen (19). To assess the effect of WASF3 overexpression on cognitive function, we used fear conditioning, a widely employed paradigm of associative learning that is quantified by motionless (freezing) time upon re-exposure to a context or discrete cue (e.g., a tone) in association with an aversive stimulus. WASF3 Tg mice showed decreased freezing time, particularly with the cued fear conditioning paradigm (Fig. 1*I*). Because specific types of contextual and cued fear conditioning depend on different parts of the brain, these data indicated that overexpression of WASF3 in the brain could compromise behavior by affecting mitochondrial metabolism and ΔΨ_m_ that underlie neuronal signaling (20, 21).

To determine whether WASF3 overexpression affects behavioral domains beyond associative fear learning, we assessed spatial learning and memory using the Morris water maze (MWM) and evaluated spontaneous locomotor and exploratory activity using the open-field test. Although both female and male WASF3 Tg mice showed impaired performance in the fear-conditioning test (*SI Appendix*, Fig. S1), only female WASF3 Tg mice exhibited deficits in the MWM, as indicated by increased escape latency during training and fewer entries into the target platform zone after training (*SI Appendix*, Fig. S2). In the open-field test, male WASF3 Tg mice exhibited significantly reduced spontaneous locomotor and exploratory activity compared with WT controls, whereas female WASF3 Tg mice did not differ significantly from their WT counterparts (*SI Appendix*, Fig. S3). These findings indicated that WASF3 overexpression can cause sex-dependent alterations in spatial learning and exploratory activity. It should also be noted that the impaired MWM performance of female WASF3 Tg mice is unlikely to be explained by locomotor deficits as they showed normal activity on open-field testing.

### WASF3 Modulates Brain Metabolism and Stress-Response Signaling.

**Fig. 2.**
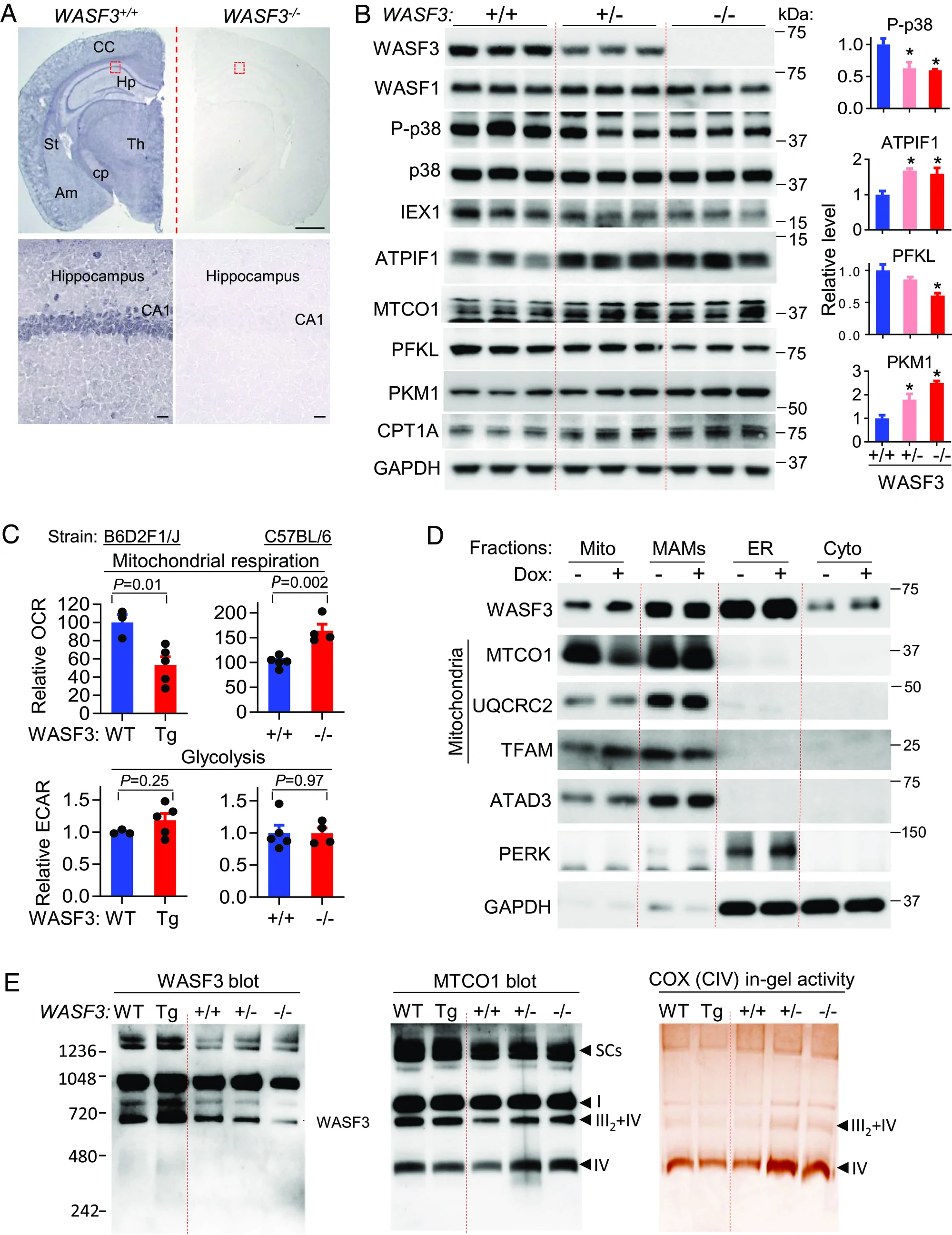
WASF3 is widely expressed in the brain with regional variation and regulates mitochondrial function. (*A*) Immunohistochemistry of representative coronal sections of *WASF3^+/+^* and *WASF3^−/−^* mouse brains mounted side-by-side on the same glass slide (*Upper* panel) to ensure parallel processing for direct comparison of antibody staining intensity (low magnification, 1.25× objective lens). Red dashed line separates the two genotypes. *Lower* panels show higher magnification (40×) of the hippocampal CA1 layer indicated by the red boxes. Cerebral cortex (CC), hippocampus (Hp), striatum (St), thalamus (Th), cerebellar peduncle (cp), amygdala (Am), and CA1 hippocampal pyramidal neurons are labeled. (Scale bars, 1,000 µm and 20 µm for low and high magnifications, respectively.) (*B*) Immunoblots of *WASF3^+/+^*, *WASF3^+/−^*, and *WASF3^−/−^* mouse hippocampal tissue lysates with quantification of the indicated proteins (right bar graphs) (n = 3). (*C*) Oxygen consumption rate (OCR, respiration) and extracellular acidification rate (ECAR, glycolysis) of whole hippocampi measured under aglycemic conditions. Note the use of two different genetic strains of mice (n = 3 to 5). (*D*) Immunoblots of subcellularly fractionated whole brain tissue of WT mice with (+) or without (−) doxorubicin (Dox) treatment. Mitochondria (Mito); mitochondria-associated membranes (MAMs); ER; cytoplasm (Cyto). (*E*) Immunoblots with WASF3 and MTCO1 antibodies and in-gel respiratory complex IV (cytochrome c oxidase) activity assay of purified brain mitochondria solubilized in 1% digitonin. Mice of the indicated genotypes were age and sex-matched. Note that WT and WASF3 Tg mice are of the B6D2F1/J strain while the *WASF3^+/+^*, *WASF3^+/−^*, and *WASF3^−/−^* mice are of the C57BL/6 strain. Values are mean ± SE. Statistical difference by one-way ANOVA statistics (*B*) and two-tailed Student’s *t* test (*C*). *P < 0.05 compared with *WASF3^+/+^*.

To further examine its physiological function, we disrupted the *WASF3* gene in mice using CRISPR/Cas9 technology (*WASF3^−/−^*) to avoid the potential neurotoxicity of Cre recombinase (22). As previously reported for the floxed *WASF3* knockout (KO) mouse (CMV-Cre *WASF3^fl/fl^*) (23), we also did not observe any overt developmental or morphological abnormalities in the *WASF3^−/−^* mouse. Immunohistochemical staining of coronal brain sections of *WASF3^+/+^* (WT) and *WASF3^−/−^* (KO) mice mounted side-by-side on the same glass slide for parallel processing revealed specific WASF3 immunoreactivity signals in WT that were lacking in the KO sections (Fig. 2 *A*, *Upper*). WASF3 protein was widely expressed in the brain, but there were regional variations in its levels including in those areas associated with fear conditioning such as the amygdala, hippocampus, and cerebellum (Fig. 2 *A*, *Upper*, and *SI Appendix*, Fig. S4). Higher magnification of WT hippocampus and cerebellum revealed strong WASF3 signals in the pyramidal neurons and Purkinje cells, respectively, that were absent in KO, demonstrating specificity of the immunolocalizations (Fig. 2 *A*, *Lower*, and *SI Appendix*, Fig. S4). Together, these data indicated that WASF3 is enriched in neurons that serve critical functions in signal integration.

**Fig. 3.**
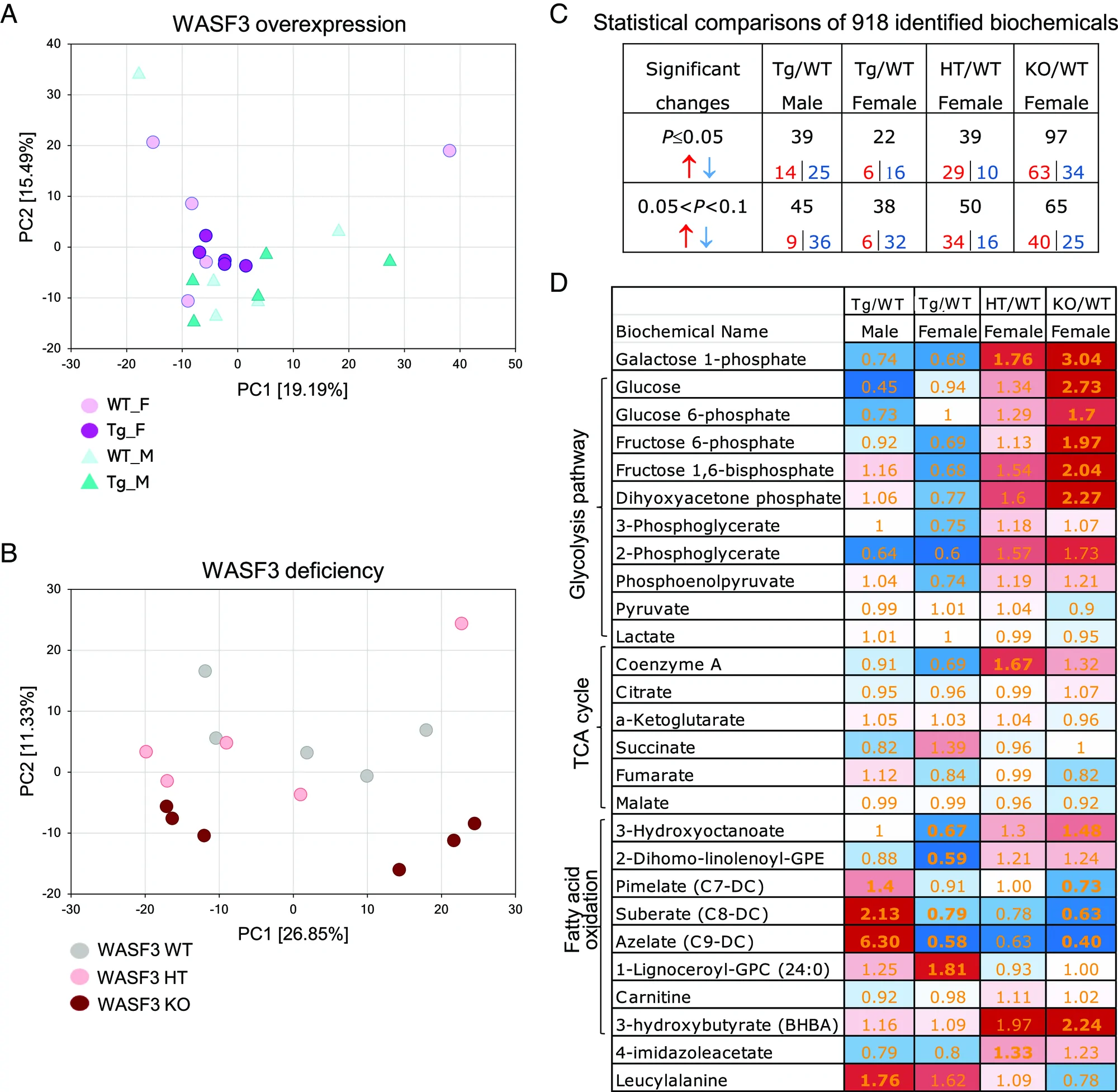
Hippocampal metabolomic profiling reveals the inhibitory effects of WASF3 on oxidative metabolism. (*A*) Principal component analysis (PCA) of mass-normalized hippocampal metabolite profiles by WASF3 genotype [WT vs. transgenic (Tg)] and by sex [female (F) vs. male (M)]. [ ] indicates % variance of the principal components. (*B*) PCA of mass-normalized hippocampal metabolite profiles of female *WASF3^+/+^* (WT, gray), *WASF3^+/−^* (HT, pink), and *WASF3^−/−^*(KO, maroon) mice. (*C*) The number of significantly increased or decreased hippocampal metabolites (out of 918 biochemicals identified) for each indicated genotype comparison is shown in red or blue, respectively. (*D*) Metabolites altered by WASF3 genotype, including some involved in glycolysis and mitochondrial metabolism, are listed in the table. Significant changes are shown in bold numbers for the indicated genotype comparison. Tg cohorts comprised of ~6 mo old male or female mice (n = 5); WT, HT, and KO cohorts comprised of ~12 wk old females (n = 5-6). Statistical difference by Welch’s two-sample *t* test (*C* and *D*).

Immunoblotting of hippocampal and cerebral cortical tissues confirmed a genotype-dependent reduction in basal WASF3 protein expression but not of its related family member WASF1 in the WASF3-deficient mice (Fig. 2*B* and *SI Appendix*, Fig. S5). Consistent with our previous finding (6), WASF3 deficiency was associated with decreased phosphorylation of p38 kinase which can induce IEX1 with subsequent degradation of ATPIF1 (Fig. 2*B* and *SI Appendix*, Fig. S5) (12, 14). The increase in ATPIF1 and MTCO1 levels in WASF3 deficient samples confirmed the inverse correlation between WASF3 abundance and core components of the oxidative phosphorylation machinery. In contrast, the glycolysis rate-limiting enzyme phosphofructokinase liver isoform (PFKL) was decreased in WASF3-deficient samples, consistent with a metabolic shift toward oxidative metabolism and aligning with prior findings of reduced PFKL expression in the hippocampi of ATPIF1 transgenic mice that have enhanced learning (Fig. 2*B*) (18). Further supporting this metabolic reprogramming, hippocampal tissue displayed a *WASF3* allele loss-dependent increase in PKM1 expression while PKM2, which is primarily expressed in neural progenitor cells, remained unchanged (Fig. 2*B* and *SI Appendix*, Fig. S6). Interestingly, PKM2, which predominantly exists in its active tetrameric form, appeared to be further increased in the *WASF3^−/−^* samples, consistent with their increased levels of fructose 1,6-bisphosphate that is known to promote PKM2 tetramerization (Fig. 3*D* and *SI Appendix*, Fig. S6) (24).

There is growing evidence that fatty acid oxidation contributes to brain metabolism and function including memory (25, 26). Under aglycemic ex vivo brain slice conditions which have been shown to uncover deficits in mitochondrial fatty acid oxidation (27), WASF3 Tg hippocampi revealed decreased OCRs, whereas tissue from *WASF3^−/−^* mice exhibited increased OCR (Fig. 2 *C*, *Upper*). Complementing the decreased OCR, WASF3 Tg samples showed a trend of increased ECAR, suggestive of a compensatory increase in glycolysis albeit under aglycemic conditions (Fig. 2 *C*, *Lower*). These results indicated that WASF3 can shift the balance between oxidative metabolism and glycolysis in the brain, in part, by its regulation of respiratory complex IV (6).

To gain further insights into WASF3 regulation of the mitochondria, we performed fractionation of whole brain tissue to determine the subcellular localization of WASF3. Immunoblotting confirmed enrichment of subcellular markers in their corresponding compartments: mitochondria (MTCO1, UQCRC2, TFAM); mitochondria-associated membranes (MAMs) (ATAD3); ER (PERK); and cytosol (GAPDH) (Fig. 2*D*) (28). Surprisingly, WASF3 partitioned predominantly in the ER, but it was also clearly detected in the mitochondrial and ATAD3-positive MAMs fractions, positioning WASF3 in the ER–mitochondrial interface. Considering the central role of MAMs in coordinating various cellular processes such as lipid metabolism, Ca^2+^ homeostasis and the unfolded protein response (UPR), this subcellular localization of WASF3 suggested that it could impact cognitive function by affecting other processes in addition to mitochondrial respiration.

There are no well-established models of ME/CFS, so we induced ER stress and WASF3 in the brain by using doxorubicin (Dox), a widely used chemotherapeutic agent that has also been associated with cognitive impairment (“chemo fog”) (29). Notably, Dox treatment increased the ER stress marker PERK with an accompanying increase in WASF3 and decrease in mitochondrial MTCO1 (Fig. 2*D*). Dox treatment also induced ER stress and WASF3 across other parts of the brain with reciprocal decreases in MTCO1 levels (*SI Appendix*, Fig. S7). As previously reported in skeletal muscle using blue-native PAGE analysis (6), WASF3 co-migrated with the mitochondrial respiratory supercomplex (SC) CIII₂+IV, and dose-dependently reduced MTCO1 protein and cytochrome c oxidase (COX) enzymatic activity levels, both in the SC CIII₂+IV and CIV positions (Fig. 2*E*). Together, these findings provided a molecular link between WASF3 and respiratory complex assembly and activity in the brain.

To examine the effect of WASF3 on global metabolism, whole hippocampi were dissected from WT, WASF3 Tg, heterozygous *WASF3^+/−^* (HT), and homozygous *WASF3^−/−^* (KO) mice and subjected to metabolomic profiling. PCA of the WT and WASF3 Tg pair revealed a modest, sex-dependent separation (Fig. 3*A*). Female WASF3 Tg samples were more tightly clustered and separated from their WT counterparts, whereas male WT and WASF3 Tg samples showed greater overlap and metabolic variability in response to WASF3 overexpression. In the WASF3 deficient cohort, the HT and KO female mice showed clearer group separation, with distinct clustering along principal component 2 (PC2, y-axis) (Fig. 3*B*). WT samples (gray) generally exhibited the highest PC2 values while the KO samples (maroon) were the lowest with HT (pink) in an intermediate position, indicating a *WASF3* gene dose-dependent effect on brain metabolism.

Compared with WT, there was a general trend of relatively decreased metabolites in the WASF3 overexpressing Tg brain tissue, whereas this pattern was reversed in the WASF3 deficient state (Fig. 3*C*). This trend was particularly evident for glycolytic metabolites in female WASF3 Tg mice which exhibited depletion of a key glycolytic intermediate fructose 1,6-bisphosphate (0.68-fold of WT) (Fig. 3*D*). One explanation for the relatively lower steady-state level of this rate-limiting metabolite could be increased glycolytic flux due to impaired oxidative phosphorylation and subsequent depletion of its substrates or intermediates. Consistent with this notion, WASF3 deficient samples showed higher levels of glycolytic intermediates in a WASF3 dose-dependent manner, possibly due to their decreased utilization in a setting of higher mitochondrial activity. This interpretation was supported by elevated levels of the tricarboxylic acid (TCA) cycle entry coenzyme A (CoA) in the WASF deficient state (1.32 KO/WT) while being reduced in WASF3 overexpressing hippocampi (0.69 Tg/WT). The TCA intermediates remained largely unchanged with the exception of succinate, a four-carbon (C4) dicarboxylic acid previously reported to be elevated in ME/CFS (30).

A number of studies have reported abnormalities in fatty acid metabolism and homeostasis in ME/CFS (30–33). In line with some of these studies, female WASF3 Tg mice exhibited reduced levels of β-oxidation and membrane lipid metabolites (e.g., 3-hydroxyoctanoate and 2-dihomo-linolenoyl GPE) while, in WASF3 deficient mice, they were increased along with the key β-oxidation ketone body β-hydroxybutyrate (BHBA) (Fig. 3*D*). Furthermore, Tg mice displayed elevated levels of the very-long-chain lysophosphatidylcholine 1-lignoceroyl-GPC (24:0), expected to accumulate with dysfunction of the peroxisome (Fig. 3*D*). Peroxisomes are necessary for very-long chain (C22-C30) fatty acid oxidation and have been suggested to be abnormal in ME/CFS (30). In addition to succinate, there was accumulation of medium chain dicarboxylic fatty acids (C7 pimelate, C8 suberate, C9 azelate) in male Tg mice, also consistent with β-oxidation impairment and their reported elevation in ME/CFS patients (30). These changes in FAO may underlie the impaired fear conditioning responses seen in WASF3 overexpressing mice given the essential role of lipid metabolism in brain function (26, 34). Additionally, WASF3 caused reciprocal changes in the levels of 4-imidazoleacetate (IAA) and leucylalanine (Fig. 3*D*). The neurotransmitter γ-aminobutyric acid (GABA) plays an important role in the fear conditioning circuitry, so it was notable that WASF3 specifically impacted the levels of IAA, a histamine-derived metabolite that can act as a potent endogenous GABA receptor agonist (Fig. 3*D*) (20, 21, 35).

Collectively, our metabolomic profiling reveals the influence of WASF3 as a negative regulator of mitochondrial activity in the brain. WASF3 overexpression appears to promote a previously described “hypometabolic syndrome” state in ME/CFS characterized by depletion of glucose and glycolytic intermediates and decreased lipid metabolism (31). These features are consistent with mitochondrial impairment and also appear to have a sex-dependent effect. In contrast, reducing WASF3 levels increases the steady-state levels of various metabolites, consistent with improved energy metabolism and enhanced mitochondrial function.

### WASF3 Downregulation Protects Against Cognitive Impairment.

**Fig. 4.**
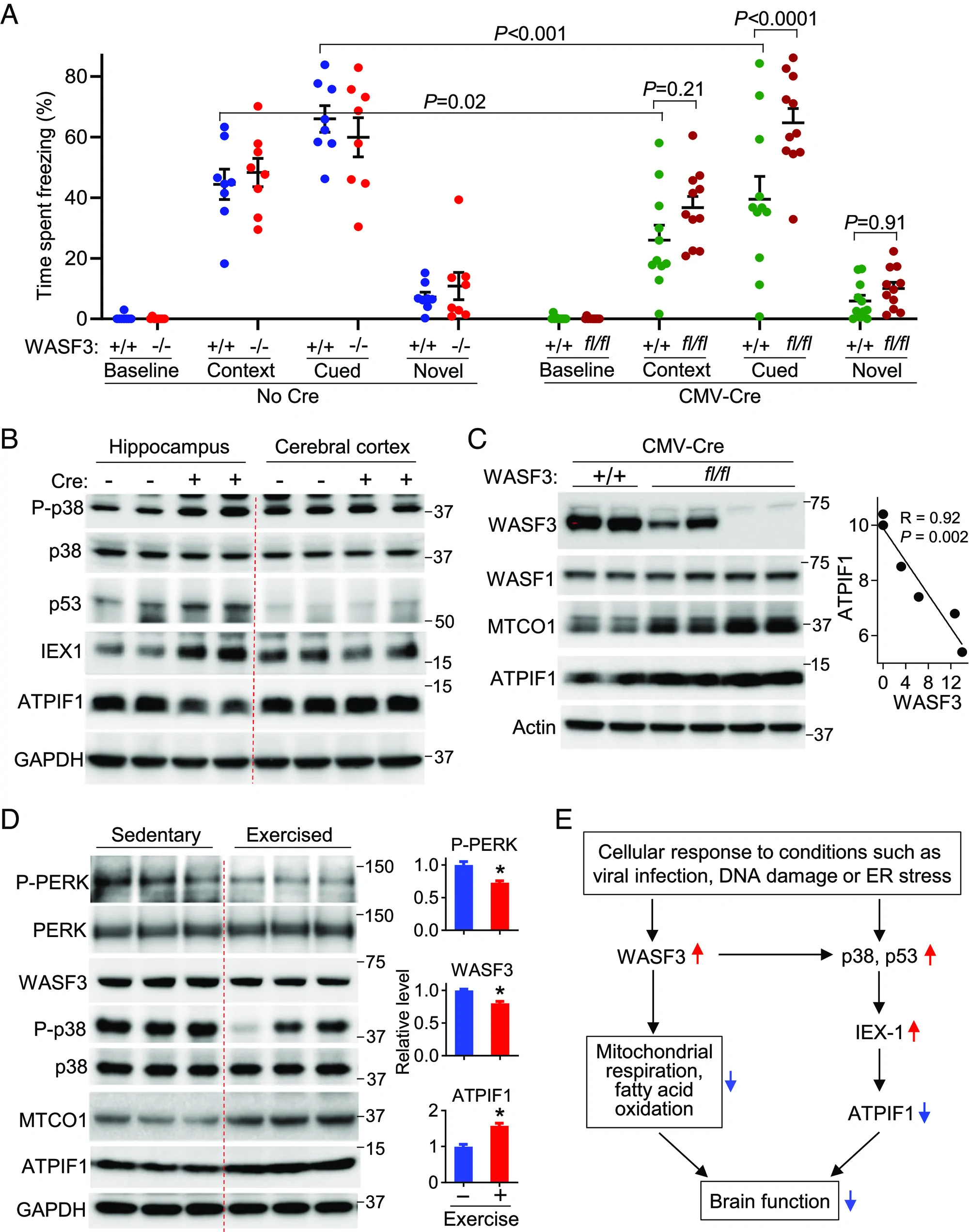
Lowering WASF3 levels protects against cognitive impairment. (*A*) Freezing times of *WASF3^−/−^* (CRISPR/Cas9-mediated knockout) and CMV-Cre *WASF3^fl/fl^* (Cre-mediated WASF3 knockout) mice and their corresponding control mice (WT without or with CMV-Cre) were measured by cued and contextual fear conditioning to assess associative learning. All mice were of the C57BL/6 strain (~4-mo-old females). (*B*) Immunoblots of some cellular stress signaling proteins and ATPIF1 in brain lysates from WT and CMV-Cre mice. (*C*) Immunoblots of mitochondrial protein MTCO1 and ATPIF1 in the cerebral cortex of CMV-Cre mice without (+/+) or with variable deletion of the floxed *WASF3* allele (*WASF3^fl/fl^*). Note the strong inverse correlation between WASF3 and ATPIF1 protein levels (*Right* panel), and the unchanged expression of family member WASF1 which serves as a negative control. (*D*) Immunoblots of whole-brain lysates of control sedentary and exercised (8 wk of voluntary wheel running) ~1-y-old WT male mice. Quantifications of the indicated proteins are shown (right bar graphs) (n = 3). (*E*) Schematic summary illustrating how cellular stress signaling and WASF3 increases can lead to mitochondrial dysfunction and other metabolic changes with subsequent impairment of cognitive function. Values are mean ± SE. Statistical difference by two-way ANOVA (*A*) and two-tailed Student’s *t* test (*D*). *P < 0.01.

At the functional level of the brain, *WASF3^−/−^* mice generated using CRISPR/Cas9 (without Cre recombinase) exhibited no overt behavioral anomalies. In the fear conditioning (FC) test, *WASF3^−/−^* mice performed similarly to WT littermates, showing comparable freezing times (Fig. 4 *A*, *Left* section). In contrast, a previously published whole-body *WASF3* knockout mouse (CMV-Cre *WASF3^fl/fl^*) obtained by crossing the CMV promoter-driven Cre (CMV-Cre) mouse with *WASF3^fl/fl^* mouse displayed significantly improved cued fear conditioning compared with control mice (CMV-Cre *WASF3^+/+^*) (Fig. 4 *A*, *Right* section) (23). Notably, Cre recombinase appeared to impair FC performance: Transgenic CMV-Cre *WASF3^+/+^* mice exhibited significantly reduced freezing time compared with WT *WASF3^+/+^* mice lacking Cre as measured by either contextual or cued FC tests (Fig. 4*A*). In support of this observation, mice expressing Cre in the brain (Nestin-Cre) have been reported to show impairments in the acquisition of both contextual and cued FC (22). Remarkably, CMV-Cre *WASF3^fl/fl^* mice showed FC acquisition comparable to that of WT *WASF3^+/+^* mice without Cre, indicating that WASF3 mediates the impairment of cued FC performance by Cre (Fig. 4*A*). Taken together, these findings indicated that WASF3 deletion does not affect FC under normal conditions but, rather, mitigates the deleterious effect of Cre on cued fear conditioning.

It is well-known that Cre recombinase expression can cause off-target DNA damage and cellular stress responses often involving p53 (36). To examine the effect of constitutive Cre expression on the brain, hippocampal, and cerebral cortical tissues from CMV-Cre transgenic mice were compared with that of WT controls (*SI Appendix*, Fig. S8). Unlike the cerebral cortex, the neural stem cell–containing hippocampus of Cre-expressing mice exhibited increased phosphorylation of p38 alongside elevated levels of p53 and IEX1, indicating DNA damage and activation of cellular stress response (Fig. 4*B*). As previously seen in the setting of increased IEX1, there was significant depletion of ATPIF1 in the Cre-expressing hippocampus (Fig. 4*B*). The relationship between WASF3 and ATPIF1 was further confirmed in the CMV-Cre *WASF3^fl/fl^* mice by taking advantage of variable *WASF3* gene deletion evident by PCR genotyping (*SI Appendix*, Fig. S9). Among the individual mice with varying levels of WASF3 protein, the expression of ATPIF1, in parallel with MTCO1, was significantly and inversely correlated with WASF3 abundance, consistent with our FC test observations and prior report linking ATPIF1 levels to cognitive performance (Fig. 4*C*) (18).

We next tested the potential regulatory relationship between WASF3 and ATPIF1 in a physiologic model of exercise training which is known to downregulate ER stress (37). Regular aerobic exercise is known to enhance cognitive function and increase hippocampal volume (38), so brain tissues from control sedentary mice versus those after 8 wk of voluntary wheel running were examined. Compared with controls, exercised mice exhibited reduced levels of ER stress marker phosphorylated PERK (P-PERK), WASF3, and phosphorylated p38, with reciprocal increases in MTCO1 and ATPIF1 in their brain tissues (Fig. 4*D*). Thus, both genetic as well as physiological manipulations in our mouse models supported a mechanism in which stress-induced WASF3 mediates cognitive impairment by disrupting mitochondrial respiration and fatty acid metabolism, as well as reducing the levels of ATPIF1, a known regulator of cognitive function (Fig. 4*E*).

## Discussion

Using complementary WASF3 genetic mouse models, we have shown that WASF3, previously implicated in ME/CFS as an ER stress-induced mediator of mitochondrial dysfunction in skeletal muscle (6, 10, 39), also acts in the brain as a negative regulator of oxidative metabolism and affects cognitive function. Increased WASF3 consistently associated with downregulation of the mitochondria, metabolic shift toward glycolysis, and impaired associative learning, whereas reducing WASF3 levels increased oxidative metabolic markers and prevented cognitive decline in a setting of cellular stress. Subcellular fractionation studies localized WASF3 to the ER–mitochondrial interface which aligns with its observed stabilization by ER stress to directly regulate mitochondrial function. These findings support a model in which dysregulated WASF3 transduces cellular stress signals into mitochondrial dysfunction, contributing to the cognitive alterations observed in ME/CFS (Fig. 4*E*) (2). Our observation of the WASF3-mediated changes in fear conditioning behavior, a key component of the posttraumatic stress disorder (PTSD) associated with ME/CFS (40–42), further underscored ER stress-WASF3 signaling as a potential contributing mechanism in this disorder.

Although best known for its role in actin cytoskeleton regulation, WASF3 has previously been reported to interact with the inner mitochondrial membrane (7, 10), In the current study, we find that WASF3 is enriched in MAMs with the bulk being in the ER. This is functionally significant as MAMs is a hub for the synthesis and transport of lipids as well as Ca^2+^ homeostasis which are integral to synaptic function (43). The enrichment of WASF3 in MAM fractions therefore provides a plausible bridge between ER stress, a proposed upstream driver in ME/CFS, and mitochondrial metabolism in the central nervous system. In addition to WASF3 activating p38, its association with MAMs may serve as another inflammatory signaling pathway by which the neuroinflammation and regional changes in brain metabolism reported in ME/CFS may be mediated (44–46).

Our investigations also identified ATPIF1 as a factor through which WASF3 may modulate brain bioenergetics and function. WASF3 overexpression reduced ATPIF1 in cultured cells and in brain tissue whereas WASF3 reduction increased ATPIF1 abundance. This reciprocal relationship may be critical for cell survival, because ATPIF1 can inhibit either the synthesis of ATP or its reverse hydrolysis for maintaining ATP homeostasis or ΔΨ_m_, respectively (11). In the setting of impaired mitochondrial respiration, downregulation of ATPIF1 may be a compensatory mechanism for maintaining ΔΨ_m_ and Ca^2+^ homeostasis and preventing neuronal cell death. Mechanistically, we observed that WASF3 can activate p38 and p53, both of which have been shown to induce IEX1, thereby promoting the degradation of ATPIF1, a regulator of ROS homeostasis (6, 12, 14, 18, 47).

Mitochondrial dysfunction, particularly disruption of respiratory complex IV, could contribute to cognitive impairment through both bioenergetic and signaling mechanisms. In WASF3 Tg mice, brain WASF3 expression was modestly elevated to levels comparable to that induced by doxorubicin, a chemotherapy associated with cognitive impairment (29). Its mitochondrial activity was reduced by ~50%, resulting in basal metabolomic alterations resembling those reported in ME/CFS (Figs. 1*H*, 2*C*, and 3*D* and *SI Appendix*, Fig. S7) (31). This bioenergetic limitation would be consequential at the synapse, where ATP is required to support critical neuronal processes such as neurotransmitter regulation and synaptic plasticity. In addition to limiting ATP availability, mitochondrial dysfunction can increase mitochondrial reactive oxygen species (mtROS) production and activate p38 MAPK stress signaling, potentially disrupting synaptic function (18, 48, 49). Although the deficits observed in fear conditioning and the water maze are consistent with impaired learning and memory, alternative explanations, such as altered stress responsivity and changes in emotional or behavioral processing, cannot be excluded. Thus, mitochondrial dysfunction mediated by WASF3, or other genes (50), likely affects cognition through multiple mechanisms.

In summary, our findings broaden the biological significance of WASF3-mediated mitochondrial regulation from skeletal muscle exercise capacity to brain function, implicating WASF3 as a potential contributor to the cognitive impairment associated with ME/CFS. WASF3 overexpression produced an in vivo steady state metabolomic picture of predominantly decreased glycolytic intermediate levels and accumulation of FAO substrates in the brain, consistent with a hypometabolic syndrome as previously reported in ME/CFS (31). Interestingly, several WASF3-associated metabolomic alterations were more pronounced in female mice, paralleling the higher prevalence of ME/CFS among women. These sex-dependent metabolic changes may also be linked to the selective impairment in spatial learning and memory observed in female WASF3 Tg mice by MWM testing. These observations highlight WASF3 as a mediator of mitochondrial dysfunction that may underlie the multisystem abnormalities including cognitive impairment seen in ME/CFS or other postviral chronic fatigue syndromes such as Long COVID (51). It is also tempting to speculate that ER stress-induced WASF3 mediates the shift from oxidative to glycolytic metabolism in other conditions with similar metabolic reprogramming such as Alzheimer’s disease (27, 52). Given our current findings on WASF3 in the brain, it is now more compelling to explore whether inhibiting ER stress, as previously demonstrated in vitro (6), can ameliorate the mitochondrial dysfunction and cognitive symptoms in patients with ME/CFS.

## Materials and Methods

### Cell Culture and Reagents.

Mouse C2C12 myoblast cells (ATCC) were cultured in DMEM [high glucose (4.5 g/L) and GlutaMAX (Gibco 10566)] supplemented with 10% fetal bovine serum and penicillin–streptomycin (50 U/mL). WASF3 cDNA constructs were transduced with lentivirus or transfected using FuGENE HD transfection reagent (Promega) and selected with blasticidin or puromycin, respectively, as previously described (6).

### Real-time RT-PCR.

Real-time RT-PCR was performed as previously described using an ABI HT7900 thermal cycler (53). mRNA expression was measured by calculating the average cycle threshold (Ct) ratio relative to housekeeping gene TIF. The PCR primer sequences were as follows.

Cre:

 Forward (F):GCGGTCTGGCAGTAAAAACTATC

 Reverse (R):GTGAAACAGCATTGCTGTCACTT

TIFF:

 F:CACGTGGGCTCCAGCATT

 R:TCACCAGTCATTTCTGCCTTTG

### Mouse Models and Strains.

The generation of the WASF3 transgenic mouse (B6D2F1/J strain) has been previously described (6). The *WASF3^fl/fl^* mouse (C57BL/6 strain JAX # 033353) (23) and the CMV-Cre transgenic mouse (C57BL/6J strain JAX # 006054) were obtained from the Jackson Laboratory. The *WASF3^fl/fl^* mouse was crossed with CMV-Cre mouse to generate WASF3 floxed knockout mice (CMV-Cre *WASF3^fl/fl^*). Genotyping was performed according to the protocol provided by JAX, and the PCR primer sequences used were as follows.

Genotyping primers for WASF3 Cre recombination:

 WT *WASF3* F:TGA GCA TTC TGA CAC CGT ACA

 LoxP *WASF3* F:CTC GTG CTT TAC GGT ATC GC

 Common R:CTG TCC CTG ACA CAT GCA AC

Genotyping primers for CMV-Cre transgenic mouse:

 Cre transgene F:CAT CCA CGC TGT TTT GAC CT

 Cre transgene R:AGG CAA ATT TTG GTG TAC GG

 Internal positive control F:AGT GGC CTC TTC CAG AAA TG

 Internal positive control R:TGC GAC TGT GTC TGA TTT CC

### Generation of WASF3 Knockout Mice Using CRISPR/Cas9.

The WASF3 knockout mouse (C57BL/6 strain) was generated using CRISPR/Cas9 technology. Briefly, two sgRNAs, one cutting Exon 3 (the first coding exon, GATTGCAGCGAGCGTGCTGT) and the other one cutting Exon 9 (the second to the last exon, CTTCTCGCATCACTTATGGG) were purchased from Synthego. These sgRNAs were first incubated with Cas9 protein (IDT) to form Cas9-sgRNA RNP complexes, and then electroporated into zygotes collected from C57BL/6 mice using a Nepa21 Electroporator (Nepa Gene Co) following procedures described by (54). The electroporated zygotes were cultured overnight in KSOM medium (Millipore Sigma, #MR-101-D) at 37 °C with 6% CO_2_. Those embryos that reached two-cell stage of development were implanted into the oviducts of pseudopregnant surrogate mothers (Swiss Webster mice, Charles River). Mice born to the foster mothers were genotyped by PCR amplification using a forward primer upstream of Exon 3 (F: GACCATGACCACATATATAC) and a reverse primer downstream of Exon 9 (R: GTAACAAATGACGGACTGTA) to identify founder mice carrying a deletion spanning the majority of the *WASF3* coding region. Founders with the desired deletion were backcrossed to WT C57BL/6 mice for three generations to minimize potential off-target mutations, and the resulting heterozygous mice were used for colony expansion and subsequent experiments.

Routine genotyping was performed by PCR detection of i) the deletion allele using the Exon 3–Exon 9 primer pair (described above) and ii) an internal WT amplicon within exon 7 using primers F: CTTCTGTCTTGGCATGCT and R: GCCATCATATTCCACTCCTGG. Because small or complex edits generated by individual sgRNAs may not be detected by the flanking PCR assay, heterozygous mice were additionally confirmed by Sanger sequencing to verify the presence of one intact WT allele across the targeted region. Sequence-verified heterozygous mice were intercrossed to generate homozygous mutants, which were identified by PCR using the Exon 3 (F) and Exon 9 (R) primer pair.

### Mouse Experimental Protocols.

All mice were maintained and handled in accordance with the NHLBI Animal Care and Use Committee. To induce ER stress, mice were injected with doxorubicin (6.7 mg/kg/wk i.p. × 3 wk) and tissue harvested 48 h after the last dose. For exercise training, mice were housed in individual cages equipped with a monitored voluntary running wheel apparatus for 8 wk (Med Associates, Inc.).

Contextual learning test was performed based on a previously published protocol as follows (55). During testing, behavioral parameters—including total freezing duration, frequency, and length of freezing bouts, and latency from stimulus presentation to freezing onset—were recorded. For tone cued fear conditioning, mice were placed in a sound-attenuated conditioning chamber (W 17 cm × D 17 cm × H 25 cm) equipped with a light, speaker, and vanilla odor. The experimental room was brightly lit and included background HEPA noise. After a 120 s baseline period, animals received two auditory tones (4 kHz, 80 dB, 15 s each), each immediately followed by a 2 s foot shock (0.85 mA) delivered through the grid floor. The tone-shock pairings were separated by a 120 s intertrial interval, after which mice were returned to their home cages. Twenty-four hours later, the experimental room was dimly lit and free of background noise. Mice were reintroduced to the original chamber with a vanilla odor, and contextual freezing behavior was assessed. After 1 h, animals were placed in a novel chamber to evaluate freezing in a new context for 120 s, followed by presentation of the auditory cue to measure cue-dependent freezing. Spatial learning and memory were assessed using the Morris water maze, while spontaneous locomotor and exploratory activity were evaluated using the open field test, as previously described by the NHLBI Murine Phenotyping Core (56).

### Seahorse XF Metabolic Studies.

For measuring ex vivo whole hippocampus metabolism, the tissue samples were embedded on islet capture microplates, and their OCR and ECAR were measured using Agilent Seahorse XFe24 analyzer under aglycemic conditions with artificial cerebrospinal fluid (NaCl 120 mM, KCl 3.5 mM, CaCl_2_ 1.3 mM, MgCl_2_ 1 mM, KH_2_PO_4_ 0.4 mM, HEPES 5 mM, pH 7.4, sucrose 10 mM) as described previously (57).

### Metabolomic Analysis.

One whole hippocampus (~25 mg) was dissected from the left hemisphere of each mouse. For each experimental group, hippocampal tissue was collected from 5 to 6 mice. Samples were rapidly frozen on dry ice and shipped to Metabolon Inc. (Morrisville, NC) for untargeted metabolomic profiling and analysis using Ultra-high Performance Liquid Chromatography–Tandem Mass Spectroscopy (UPLC–MS/MS).

### Isolation of MAMs, Mitochondria, ER, and Cytosol from Mouse Brain.

MAMs, mitochondria, ER, and cytosolic fractions were isolated from mouse brain as previously described (58). Briefly, brain tissue was homogenized in ice-cold Solution A (0.32 M sucrose, 1 mM NaHCO_3_, pH 7.4, 1 mM MgCl_2_, 0.5 mM CaCl_2_) supplemented with protease and phosphatase inhibitor cocktails (Roche) using a prechilled glass Dounce homogenizer. The homogenates were cleared of nuclei, intact cells, and debris by low-speed (~800 to 1,400×*g*) centrifugation. The pellets were rehomogenized once, and the supernatants were pooled. The pooled supernatants were then centrifuged at 13,800×*g* to obtain mitochondria-enriched pellets which were again rehomogenized and centrifuged to maximize mitochondrial recovery, while the mitochondria-depleted supernatants (containing cytosol and ER) were saved on ice. The mitochondrial pellets were then resuspended in Solution B (0.32 M sucrose, 1 mM NaHCO_3_, pH 7.4, protease and phosphatase inhibitors), layered on a discontinuous sucrose gradient (3 mL each of 0.85 M, 1.0 M, and 1.2 M sucrose), and centrifuged at 82,500×*g* at 4 °C for 2 h. The resulting pellets (crude mitochondria) was resuspended in isolation medium (250 mM mannitol, 5 mM HEPES, pH 7.4, 0.5 mM EGTA, 0.1% BSA) and separated on a 30% Percoll gradient (95,000×*g*, 30 min, 4 °C) to obtain a heavy mitochondria band (lower) and a light MAMs band (upper). The lower band was diluted with isolation medium and spun at 6,300×*g* to obtain the final mitochondrial pellet. The upper band was also diluted and spun at 6,300×*g* and the resulting supernatant was then centrifuged at 100,000×*g* at 4 °C for 1 h to pellet the MAMs fraction. The saved supernatant (mitochondria-depleted) from the 13,800×*g* centrifugation step was further centrifuged at 100,000×*g* at 4 °C for 1 h to yield the ER fraction (pellet) and the cytosolic fraction (supernatant).

### Isolation of Mitochondria from C2C12 Cells.

Mitochondria were purified from C2C12 cells by differential centrifugation as previously described (6). Briefly, cells were harvested on ice, washed with ice-cold PBS, and resuspended in mitochondrial isolation buffer (250 mM sucrose, 50 mM Tris–HCl, pH 7.4, 5 mM MgCl_2_) supplemented with protease and phosphatase inhibitor cocktails (Roche). Cells were mechanically disrupted by Dounce homogenization (3 cycles of 20 strokes). Homogenates were cleared of nuclei and unbroken cells by sequential low-speed centrifugation (~800×*g* at 4 °C for 10 min). Then the mitochondria were pelleted from the resulting supernatant by centrifugation at 10,000 to 12,000×*g* at 4 °C for 10 min followed by one to two washes in isolation buffer.

### Mitochondrial Membrane Potential and Directed Mitochondrial Motion Measurements.

Mitochondria were labeled with 150 nM MitoTracker Green (Molecular Probes, Eugene, Oregon, USA) or TMRE Mitochondrial Membrane Potential Assay Kit (CAYMAN CHEMICAL, Michigan, USA) for 30 min at 37 °C and 5% CO_2_. To see the individual cells, nuclei were labeled with 10 µM Hoechst (ThermoFisher Scientific, MA). Then, the cells were washed and incubated in fresh culture medium for the imaging. Fluorescence images of the cells were acquired using a commercial Zeiss 880 Confocal laser scanning microscope (Jena, Germany) equipped with a Plan-Apochromat 63×/1.4 NA Oil immersion objective. For detecting the changes of mitochondrial membrane potential, TMRE was excited at 488 nm and the fluorescence signal was spectrally filtered by selecting a 550 to 590 nm bandpass filter. Hoechst was excited at 405 nm and the fluorescence signal was filtered by selecting a 415 to 481 nm filter.

For directed mitochondrial motion studies, several time-series images of mitochondria were collected by exciting MitoTracker Green at 488 nm and filtering the fluorescence signal by selecting a 490 to 531 nm bandpass filter. The individual frames of each series (70 frames) were collected with average pixel dwell time of 1.95 μs and no added time delays between the sequential frames. Spatiotemporal image correlation spectroscopy (STICS) analyses were performed using MATLAB R2022b (The MathWorks Inc.) routines adapted from the works of Wiseman Research group, McGill University (59, 60). These fluorescence based microscopy techniques are appropriate for the study of molecular diffusion and flow on time scales ranging from microseconds to milliseconds. The spatiotemporal autocorrelation functions were performed for different subregions of the images and the component of these correlation functions was averaged and fitted by nonlinear least squares. Each image correlation analysis was performed on at least 8 cells of each group with at least 12 distinct subregions for each cell.

### Antibodies and Immunoblotting.

Protein samples were solubilized in cold RIPA lysis buffer with protease inhibitor cocktail (Roche), resolved by Tris-glycine or Bis-Tris SDS PAGE, and transferred to Immobilon-P membrane (Millipore) for standard immunoblotting using ECL Plus Western Blotting Substrate (Pierce). The visualized proteins were quantified using ChemiDoc™ Touch Imaging System (Bio-Rad).

Antibodies utilized in this study were as follows:

Actin (mAb, A1978) (Sigma); ATAD3 (pAb, 16610-1-AP)(Proteintech); MTCO1 (mAb, ab14705), total Oxphos cocktail (mAb, ab110413), TFAM (pAb, ab131607) (Abcam); p38 (pAb, 9212), P-p38 (pT/Y) (pAb, 9211), Phospho (Thr980) PERK (pAb, 3179), PERK (mAb 3192), PKM1 (mAb, 7067), PKM2 (pAb, 3198), ATPIF1 (pAb, 8528), WASF3 (pAb, 2806), p53 (1C12) (mAb, 2524), PFKL (mAb, 89495) (Cell signaling); GAPDH (mAb, AM4300) (Ambion); IEX1 (pAb, PA520391) (Thermofisher); UQCRC2 (pAb, PA5-51152) (Invitrogen); WASF1 (mAb, sc-271507) (Santa Cruz).

### Blue Native Polyacrylamide Gel Electrophoresis (BN-PAGE).

Purified mitochondria were solubilized in NativePAGE sample buffer (Invitrogen) containing 1% digitonin at 4 °C for 1 h and then centrifuged at 20,000×*g* for 20 min. Approximately 10 µg of protein in the supernatant (containing mitochondrial complexes) were resolved in a 4 to 16% gradient NativePAGE Novex Bis-Tris gel as previously described with the following modifications (6, 61). Briefly, the in-gel COX (complex IV) activity was detected in solution with cytochrome C and diaminobenzidine (DAB). For immunoblotting, the gel, after electrophoresis, was incubated in transfer buffer supplemented with 2% SDS and 100 mM DTT for 15 min before being transferred to immobilon-PVDF membrane for immunoblotting.

To assess PKM2 oligomerization in mouse brain, hippocampal tissue was homogenized in buffer containing 20 mM Bis-Tris (pH 7.4), 125 mM caproic acid, 20 mM KCl, 2 mM EDTA, 5 mM MgCl_2_, 10% glycerol, and 2% n-dodecyl-β-D-maltoside (DDM). The homogenate was centrifuged at 14,000×*g* at 4 °C for 30 min, and the protein concentration of the resulting supernatant was determined. Equal amounts of protein were separated by native PAGE and immunoblotted with an anti-PKM2 antibody as previously described (62).

### Immunohistochemistry.

WT and *WASF3^−/−^* mice were deeply anesthetized with isoflurane and cardiac perfused with PBS followed by 0.5% paraformaldehyde in PBS. Brains were removed, frozen in optimal cutting temperature (OCT) compound, oriented accordingly, and sectioned (16 µm) using a cryostat. Following postfixation in cold methanol at −20 °C for 15 min and washing three times with PBS, sections were subjected to immunohistochemical staining using the Vectastain Elite ABC-HRP Kit (Rabbit IgG) (PK-6101, Vector Laboratories, Inc.). The sections were blocked with 1.5% goat serum at room temperature for 60 min. Primary antibody incubation was performed with rabbit polyclonal WASF3 antibody (2806, Cell Signaling) diluted (1:100) in 0.5% BSA in PBS at 4 °C overnight. Signal detection was carried out using secondary HRP antibody and diaminobenzidine substrate with nickel (SK-4100, Vector Laboratories, Inc.) according to the manufacturer’s instructions.

### Statistical Analysis.

GraphPad Prism software (version 7) was used for statistical analysis. Two-tailed unpaired Student’s *t* test was used for statistical comparison with corresponding controls unless otherwise noted.

## Supplementary Material

Appendix 01 (PDF)

## Data Availability

Primary data (metabolomics and biochemistry) have been deposited in FigShare (63).
