# Supplementary material for: Stress-inducible WASF3 regulates brain metabolism and cognitive function
Source: Proc Natl Acad Sci U S A. 2026 Oct 27;123(44):e2617198123. doi: 10.1073/pnas.2617198123 (PMC13732061; doi:10.1073/pnas.2617198123)
Supplement: Supplementary file 1 [file pnas.2617198123.sapp.pdf]

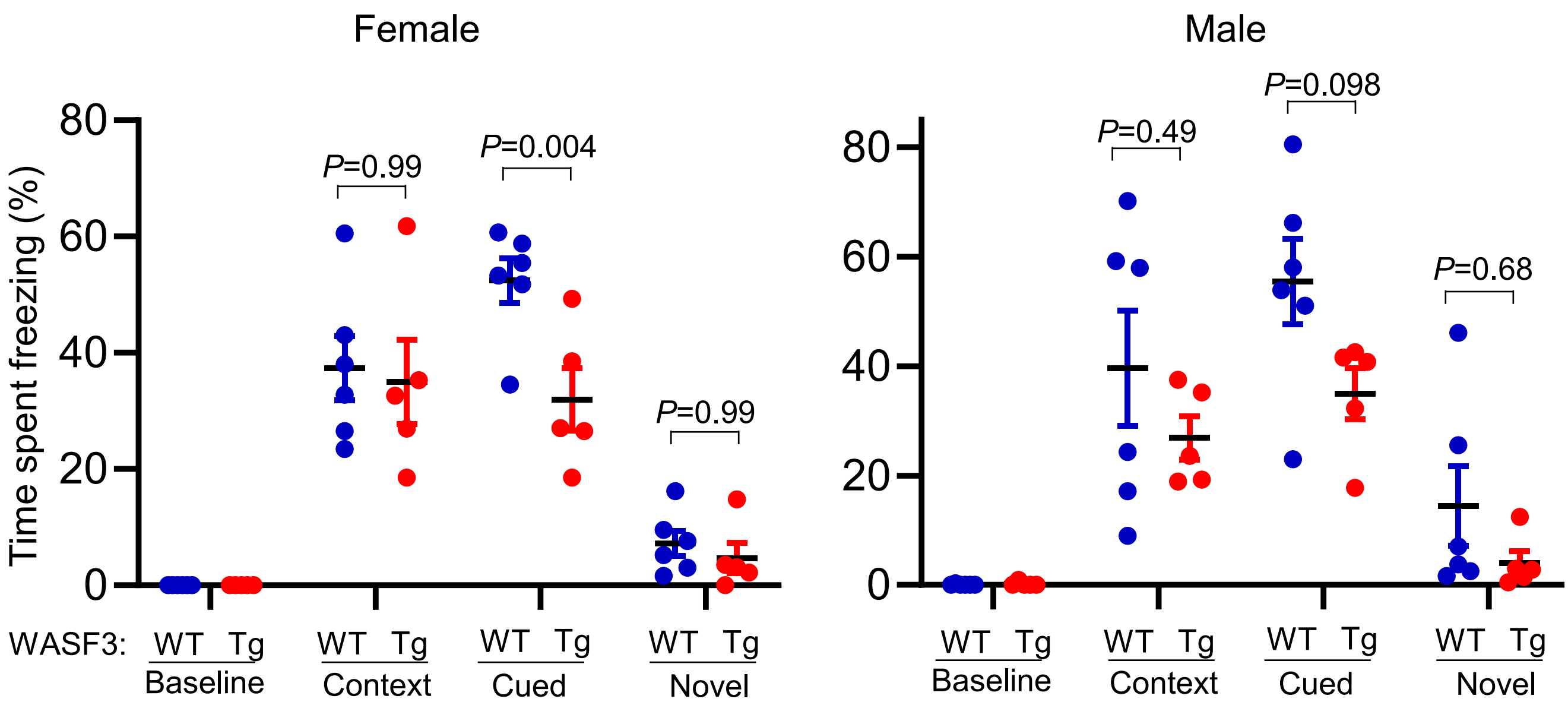

Fig. S1. Fear conditioning testing of WASF3 transgenic mice. Female WASF3 Tg mice showed significantly decreased freezing time in cued test while male mice exhibited a trend of lower freezing times. Approximately 4 month old mice were used for the test (n = 5-6). Data were analyzed by 2-way ANOVA.

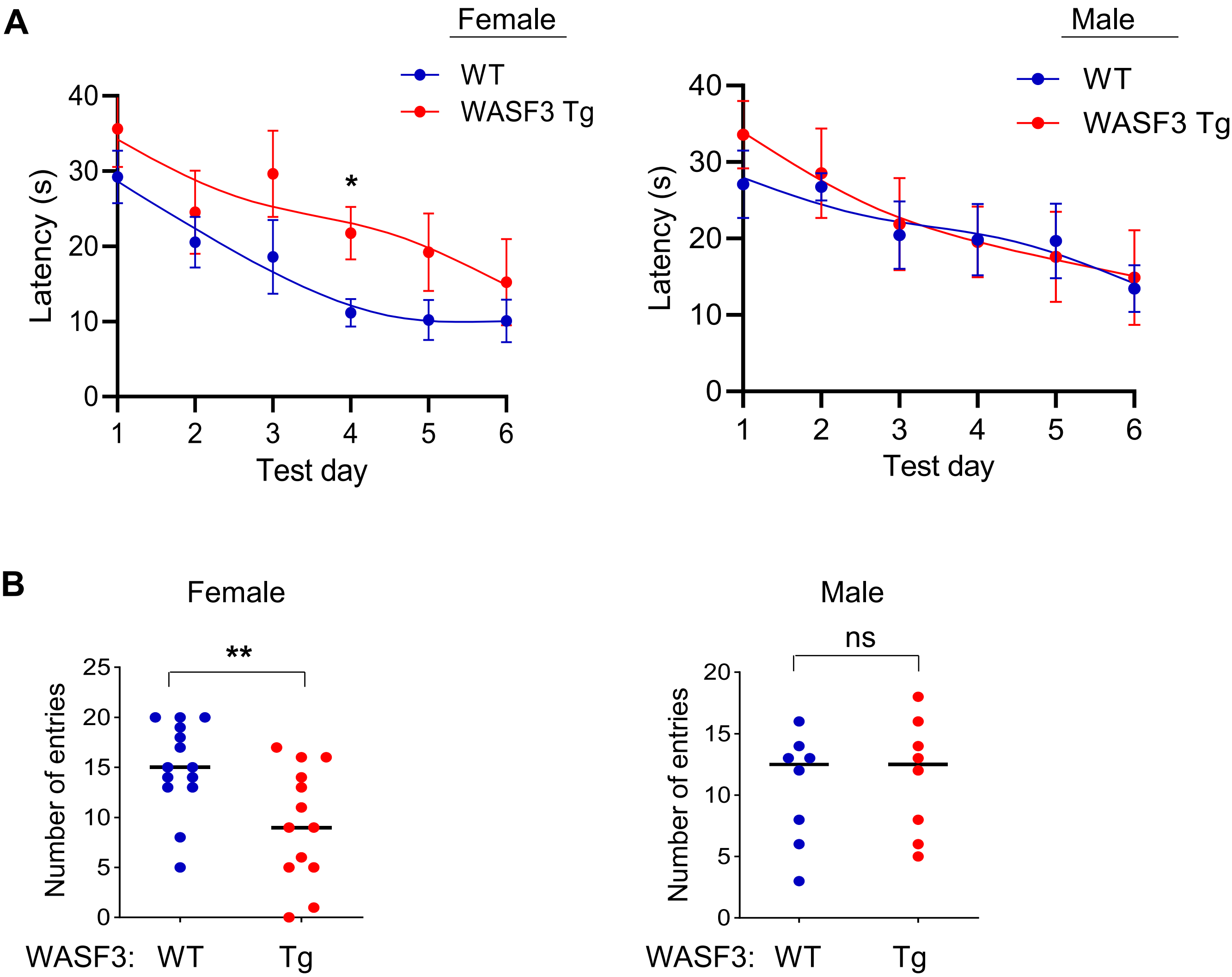

Fig. S2. Female WASF3 transgenic mice display impaired spatial learning and memory in the Morris water maze. (**A**) Escape latency, defined as the time required to locate the hidden platform, was increased in female WASF3 Tg mice over the course of training. (**B**) Number of entries into the target platform zone on the final day of the water maze test. Female WASF3 Tg mice showed fewer entries into the target zone following training. Approximately 4 month old female mice were used for the test (n = 8-14). Data were analyzed using 2-tailed Student's t test . *$P < 0.05$, **$P < 0.01$; ns, not significant.

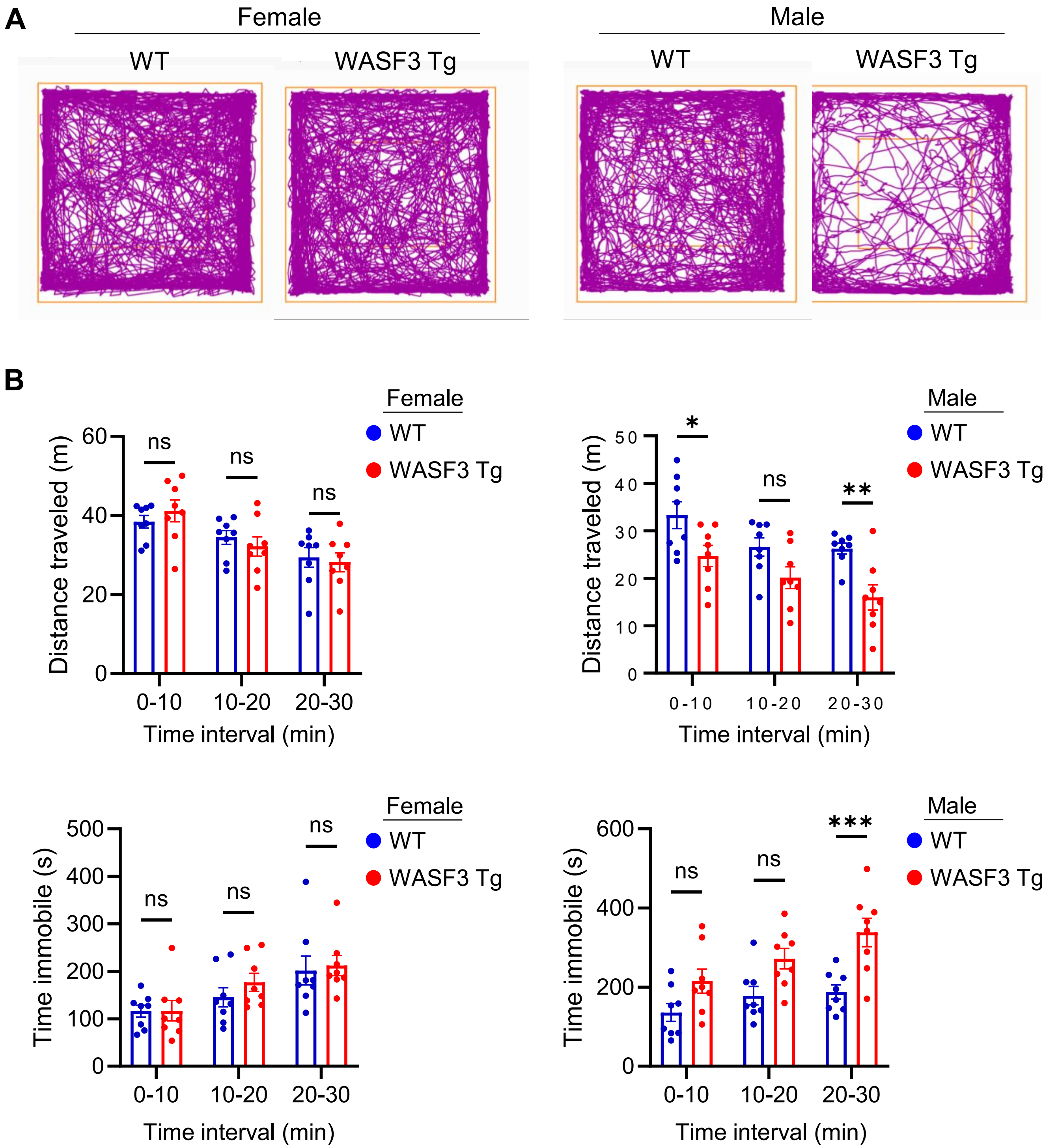

Fig. S3. Male WASF3 transgenic mice display reduced exploratory activity. (**A**) Representative track plots of WT and WASF3 Tg mice during the open field test. The positions of the mouse's center point for the entire duration of the test are shown. (**B**) Exploratory activity, measured as total distance traveled and time spent immobile during the successive time intervals, in WT and WASF3 Tg mice are shown. Approximately 4 month old mice were used for the test (n = 8). Data were analyzed by 2-way ANOVA. *$P < 0.05$, **$P < 0.01$, ***$P < 0.001$, ns, not significant.

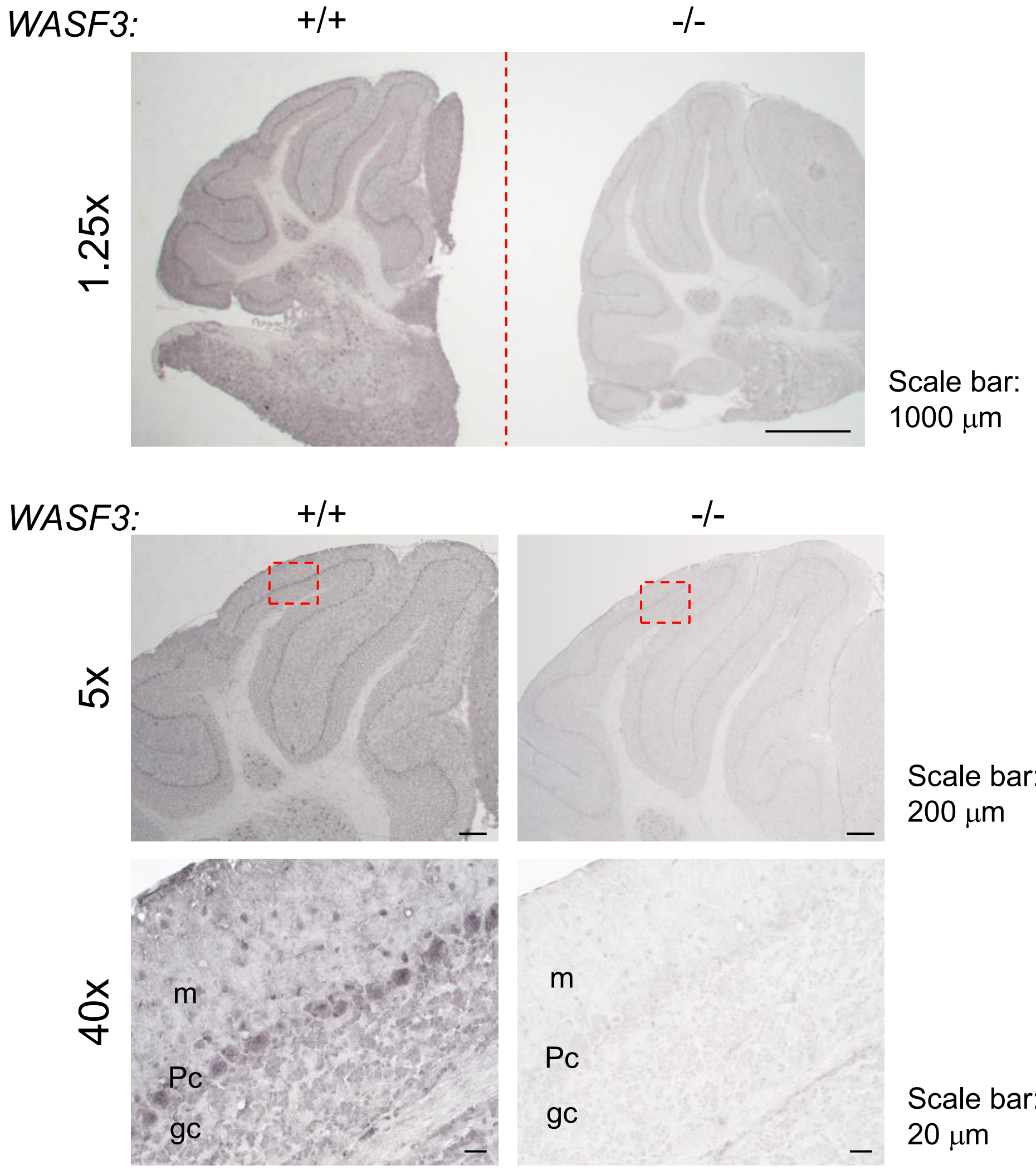

Fig. S4. WASF3 is expressed in the cerebellum.
Immunohistochemistry of representative sections of *WASF3*$^{+/+}$ and *WASF3*$^{-/-}$ mouse cerebellum mounted side-by-side on the same glass slide (upper panel) to ensure parallel processing for direct comparison of antibody staining (low magnification, 1.25x objective lens). Red dashed line separates the two genotypes. Lower panels show higher magnification (5x and 40x) of the molecular (m), Purkinje cell (Pc) and granule cell (gc) layers of the cerebellum in the indicated red boxes.

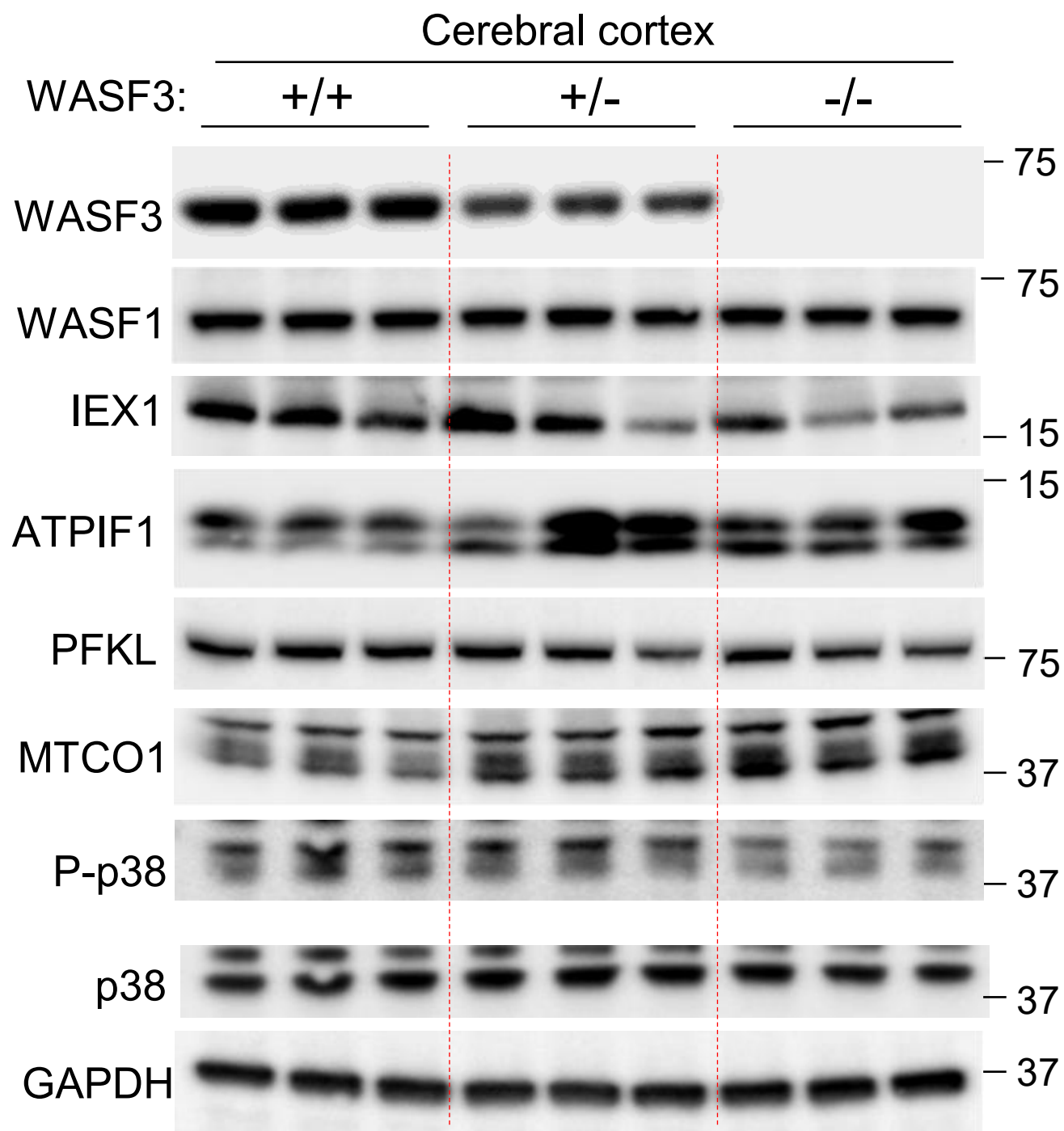

Fig. S5. WASF3 expression in the cerebral cortex. Cerebral cortical tissue of *WASF3*$^{+/+}$, *WASF3*$^{+/-}$ and *WASF3*$^{-/-}$ mice were immunoblotted with the indicated antibodies. Note the increased ATPIF1 and decreased P-p38 in WASF3-deficient samples.

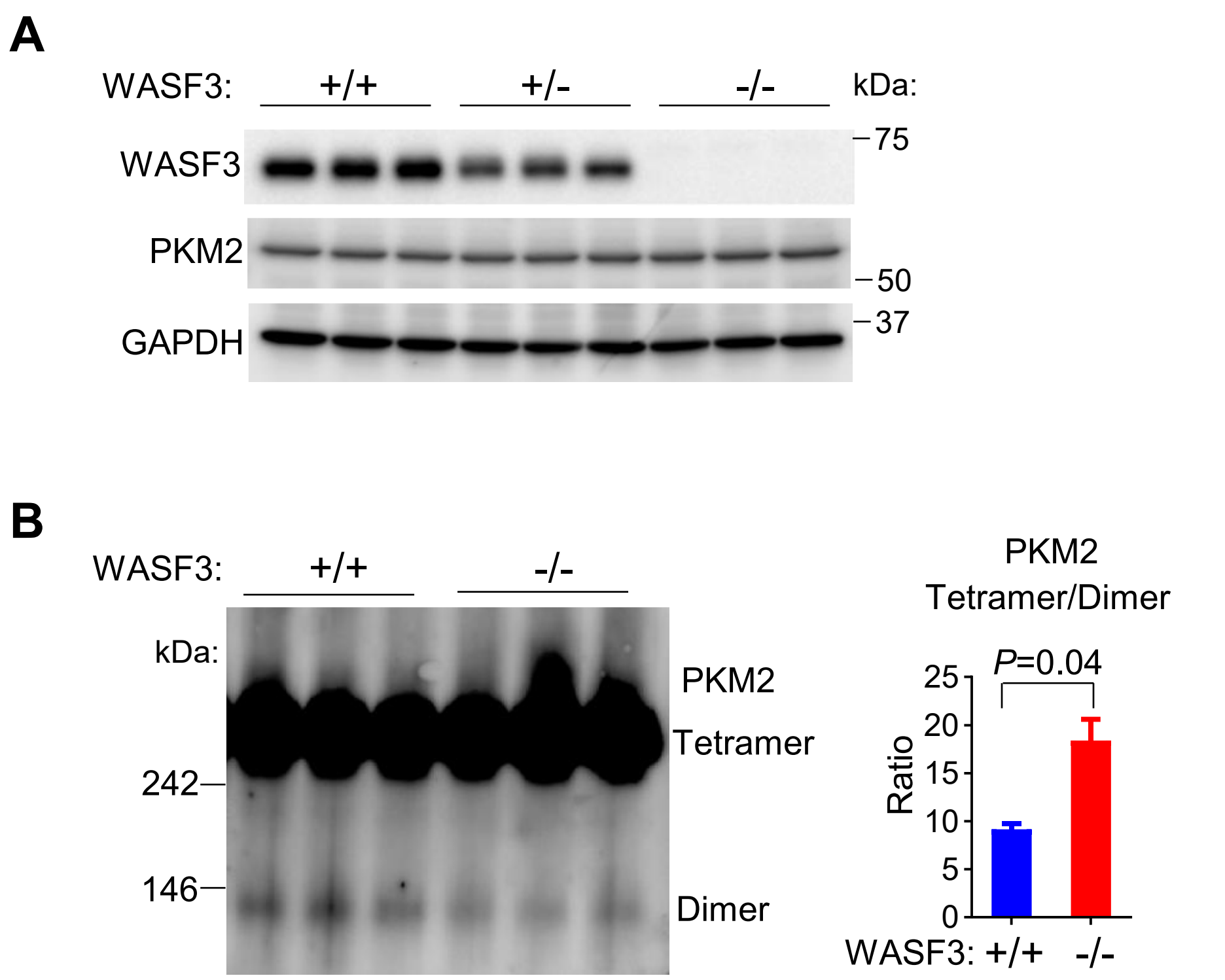

Fig. S6. Effect of WASF3 on PKM2 in mouse hippocampal tissues. (**A**) Proteins of the indicated samples were resolved by SDS-PAGE and immunoblotted with the indicated antibodies. (**B**) Proteins were resolved by blue native PAGE (BN-PAGE) and immunoblotted with an anti-PKM2 antibody. The tetramer and dimer bands of PKM2 were quantified, and their ratios are shown in the right panel (n=3). Data were analyzed using 2-tailed Student's *t* test .

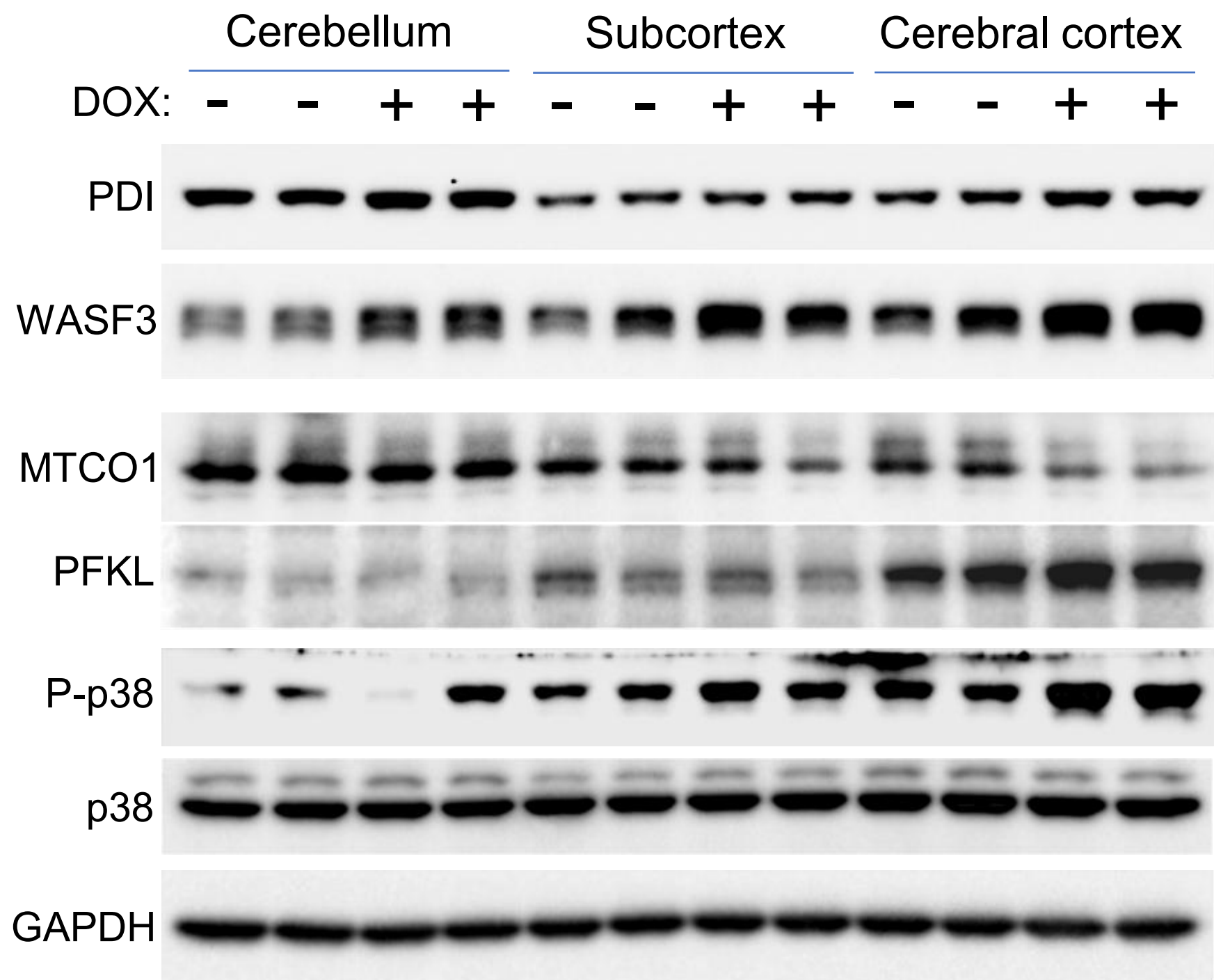

Fig. S7. Effect of doxorubicin (DOX) treatment on WASF3 and WASF3-associated molecules in brain. After treatment with Dox (6.7 mg/kg/wk i.p.) for 3 wk, the indicated parts of the mouse brain were dissected 2 d after the last dose and immunoblotted. After dissecting out the cerebral cortex, the remaining parts of the subcortical structures were also immunoblotted. PDI serves as an ER stress response marker.

A

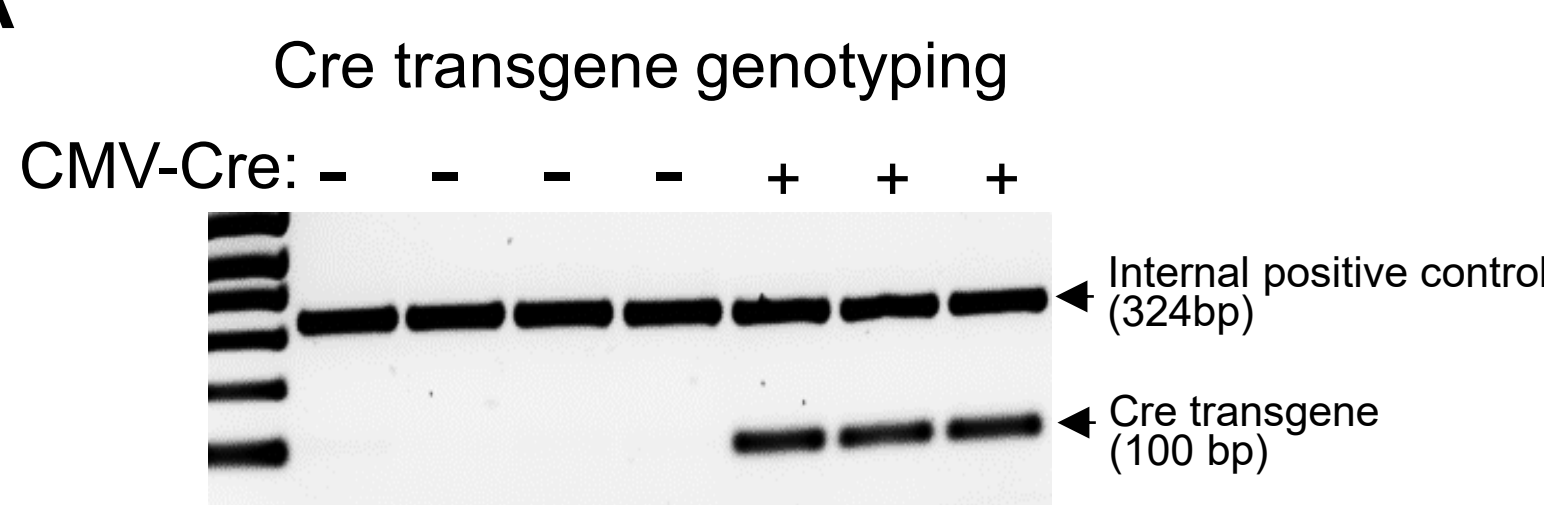

B

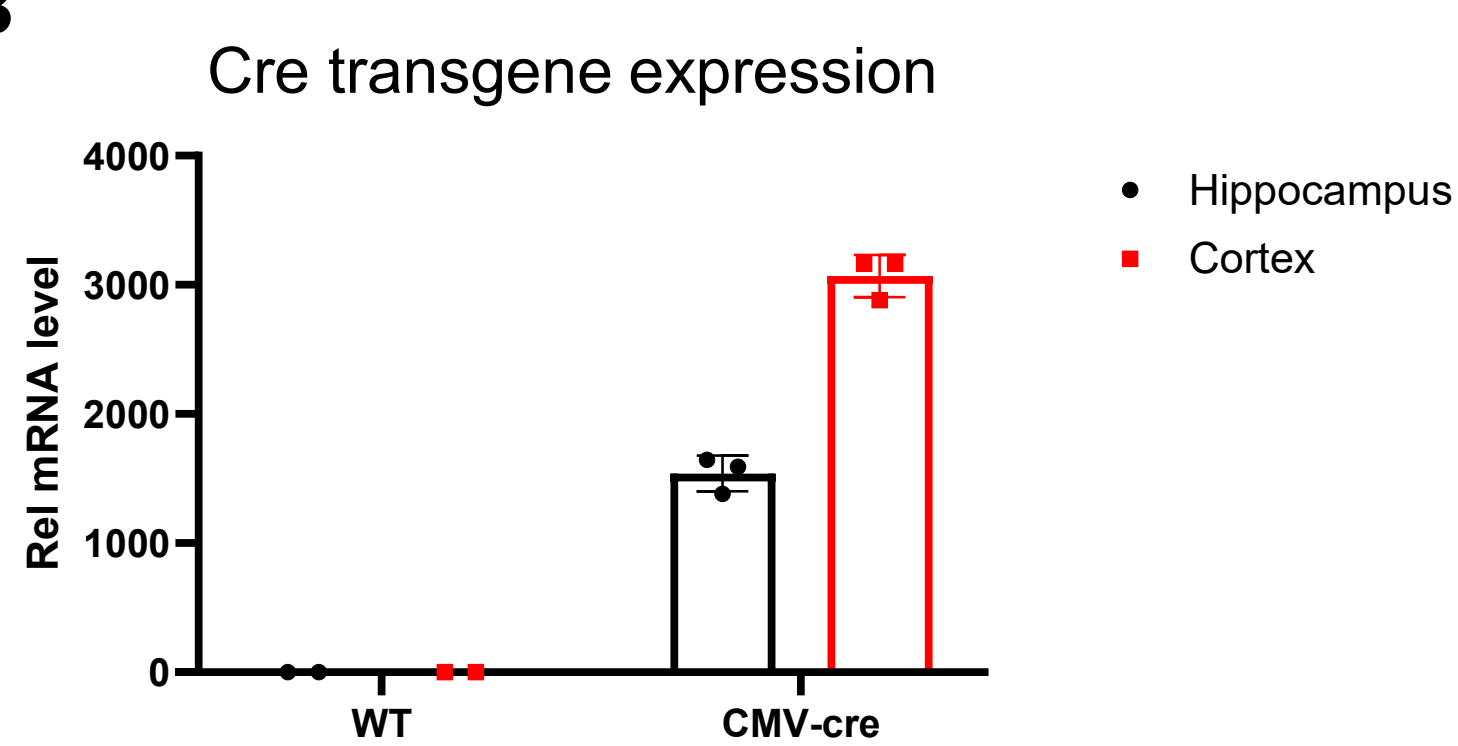

Fig. S8. Cre transgene genotyping and confirmation of its expression in the cerebral cortex and hippocampus of mouse brain.

(**A**) Representative examples of PCR-based genotyping of wild-type (**-**) versus CMV-Cre (+) transgenic mice. (**B**) Quantification of Cre transgene mRNA levels in hippocampus and cerebral cortex by real-time RT-PCR.

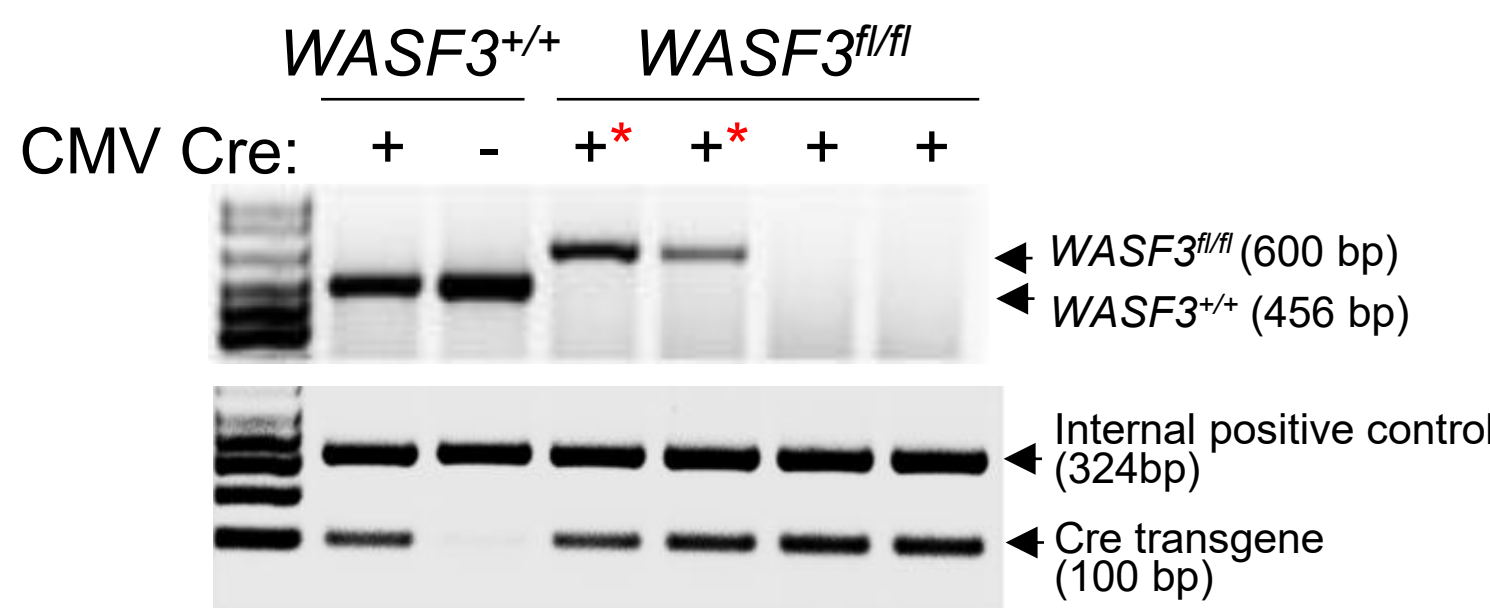

Fig. S9. A representative genotype PCR showing variable deletion of the *WASF3* gene in CMV-Cre *WASF3*$^{fl/fl}$ mice. Tail genomic DNA (gDNA) was used to detect the deletion of the loxP-flanked *WASF3* allele (*WASF3*$^{fl/fl}$, 600 bp; *WASF3*$^{+/+}$, 456 bp). Red asterices indicate partial deletion of the floxed *WASF3* allele. An internal positive control (324 bp) for gDNA and the Cre transgene (100 bp) fragments of the corresponding samples are shown.
